# Refractory hyponatremia mimicking worsening depression after suspected dimethoate exposure, influenza B infection, and venlafaxine up-titration: a case report

**DOI:** 10.1186/s12888-026-08009-w

**Published:** 2026-03-28

**Authors:** Conghao Sun, Guilan Sun, Ke Chen, Zheli Chen, Zhixing Shen, Yu Fang

**Affiliations:** Department of Psychiatry, The Third People’s Hospital of Huzhou Municipal, The Affiliated Hospital of Huzhou University, Zhejiang, China

**Keywords:** Organophosphate exposure, Dimethoate, Hyponatremia, Relative hypocortisolemia, HPA axis, Venlafaxine, Influenza B, Second-hit model, Case report

## Abstract

**Background:**

Endocrine disturbances after organophosphate exposure are rarely emphasized in routine psychiatric care. In older inpatients, severe hyponatremia is often attributed to antidepressant-induced SIADH, which may delay recognition of relative hypocortisolemia. We report a hypothesis-generating case supporting a multifactorial ‘second-hit’ model.

**Case presentation:**

A 67-year-old woman with long-standing major depressive disorder attempted suicide after an argument with family members by ingesting a suspected dimethoate emulsion (half a bottle found at the scene), approximately 20 estazolam tablets, and a small amount of detergent. She was transferred to a psychiatric ward for presumed depressive exacerbation. On day 1, serum sodium was 141.0 mmol/L (reference 137.0-147.0) but 8 AM cortisol was low at 112.4 nmol/L (reference 166-507). Influenza B was confirmed on day 2. After venlafaxine XR was initiated (75 mg/day) and up-titrated to 150 mg/day on day 4 because of apparent non-response, sodium progressively declined to 125.0 mmol/L on day 7, continued to fall to 120.2 mmol/L on day 8 and reached a nadir of 119.4 mmol/L on day 9, and remained low on day 10 (123.9 mmol/L) despite supplementation and fluid restriction. Repeat 8 AM cortisol was 122.0 nmol/L with an inappropriately normal ACTH of 24.4 pg/mL (reference 7.2-63.3) given systemic inflammation. Brain CT, adrenal ultrasound, and thyroid function were unremarkable. Urine sodium/osmolality and plasma osmolality were not measured. Oral prednisone acetate 5 mg twice daily was started on day 11 while venlafaxine was continued. Sodium rose to 130.0 mmol/L on day 12 and normalized by day 16 (141.0 mmol/L), accompanied by rapid improvement in appetite, spontaneous speech, and psychomotor activity.

**Conclusions:**

Causality cannot be definitively established. However, this case highlights that medically stressed psychiatric inpatients may develop refractory hyponatremia from interacting factors (infection, SNRI up-titration, and relative HPA-axis insufficiency). Early-morning cortisol assessment should be considered when hyponatremia co-occurs with apparent antidepressant non-response, particularly in older, low-BMI patients.

## Background

Organophosphate (OP) pesticides remain a frequent method of self-harm in many regions. Beyond acute cholinergic toxicity, OP exposure has been associated with transient endocrine abnormalities, though clinical evidence is limited [1–3]. In psychiatric wards, hyponatremia is common and often attributed to SIADH related to infection or antidepressants. Glucocorticoid deficiency can reduce free-water clearance and closely mimic depressive worsening through fatigue, anorexia, and psychomotor retardation [4–6]. We describe a case in which suspected OP exposure and systemic inflammation preceded refractory hyponatremia, while influenza B infection and venlafaxine up-titration may have acted as ‘second hits’.

## Case presentation

A 67-year-old female (height 150 cm, weight 40 kg at admission; BMI 17.8 kg/m^2) with a 10-year history of major depressive disorder attempted suicide after an argument with family members. Family members found half a bottle of dimethoate emulsion (brand name ‘Leku’) at the scene; the ingested dose was unknown. Serum cholinesterase was 4100 U/L, at the lower limit of our laboratory reference range (4000–11000 U/L), and overt cholinergic signs were not prominent, so exposure was treated as suspected. She also reportedly ingested ~ 20 tablets of estazolam and a small amount of household detergent. Depressive symptoms were reportedly stable during the 1–2 weeks preceding the attempt.

**Fig. 1:**
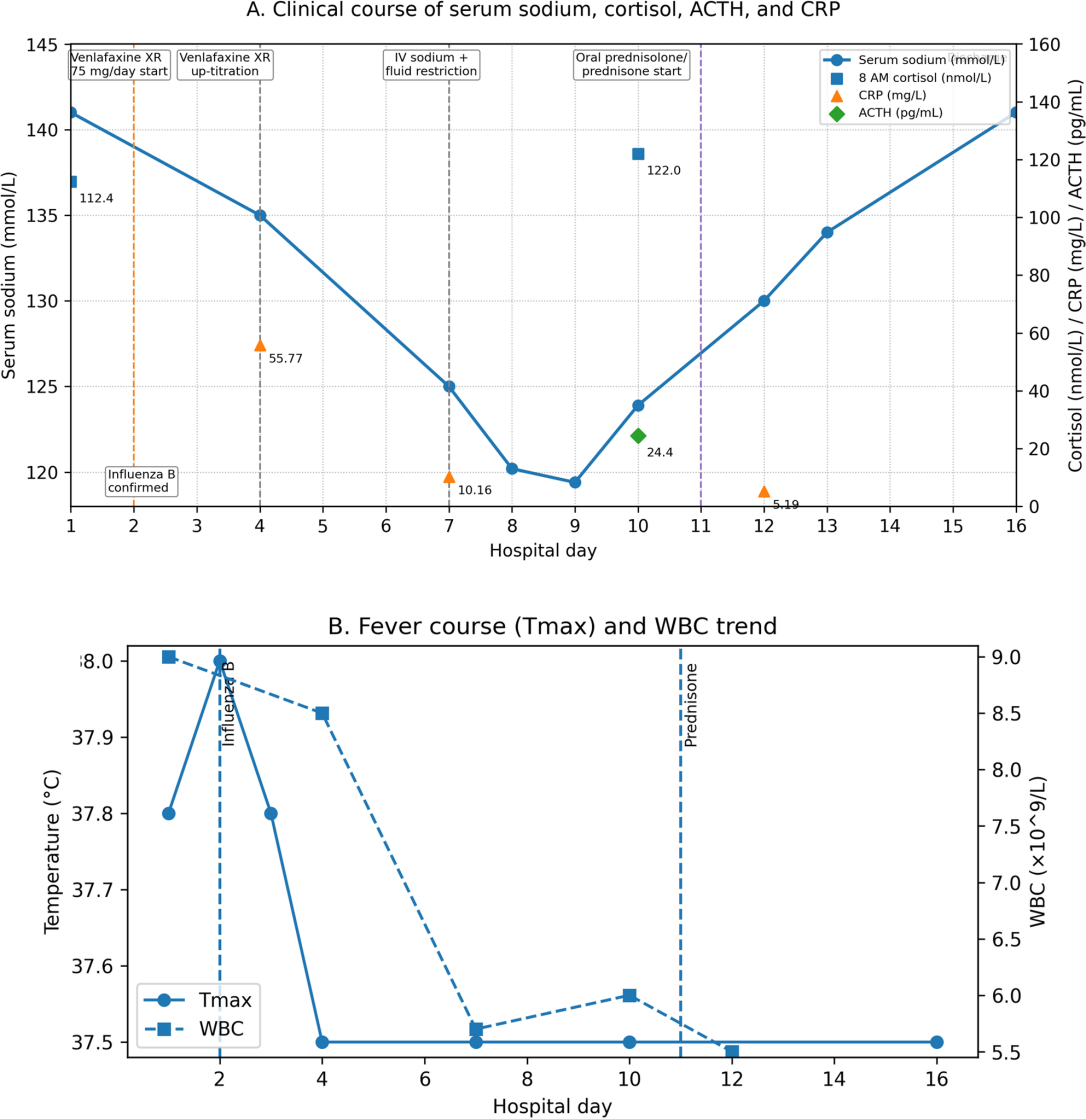
Timeline of clinical course and laboratory trends. Timeline of clinical course and laboratory trends. Panel A shows serum sodium (left y-axis) with cortisol (8 AM) and CRP (right y-axis) and key interventions (influenza B confirmation on day 2, venlafaxine XR up-titration on day 4, prednisone initiation on day 11). Panel B shows fever course (Tmax) and WBC trend over the same period

On arrival, she had low-grade fever (Tmax 37.8–38.0 °C on days 1–2). Pupils were ~ 3.5 mm; nausea was noted, but there was no vomiting/diarrhea, diaphoresis, bronchial hypersecretion, or fasciculations recorded. Atropine and pralidoxime were not administered. Baseline hemoglobin was mildly reduced (104 g/L). Key laboratory values and interventions are summarized in Table 1, and their temporal relationships (including fever/WBC trends) are depicted in Fig. 1.

She was admitted to a psychiatric ward for presumed acute depressive exacerbation with marked apathy, reduced speech and activity, and poor intake. Venlafaxine XR was initiated at 75 mg/day on day 1 and increased to 150 mg/day on day 4 due to apparent non-response. Serum sodium was normal on day 1 (141.0 mmol/L) but fell to 135.0 mmol/L on day 4 and to 125.0 mmol/L on day 7. Despite sodium supplementation with fluid restriction, sodium continued to decline to 120.2 mmol/L on day 8 and 119.4 mmol/L on day 9, and remained low on day 10 (123.9 mmol/L). Fasting glucose (4.5–5.3 mmol/L) and triglycerides (0.73–1.15 mmol/L) were within normal ranges, making pseudohyponatremia unlikely. Thyroid function was normal (T3 1.85, FT3 5.30, TSH 1.22).

Morning cortisol was low on day 1 (112.4 nmol/L) and remained low on day 10 (122.0 nmol/L) with ACTH 24.4 pg/mL, which was considered inappropriately normal in the setting of systemic inflammation (CRP 55.77 mg/L on day 4). Brain CT and adrenal ultrasonography were unremarkable. Urine sodium/osmolality, plasma osmolality, and ACTH stimulation testing were not obtained. Multidisciplinary consultation favored relative HPA-axis insufficiency/CIRCI as a contributor to refractory hyponatremia. Oral prednisone acetate 5 mg twice daily was started on day 11, and venlafaxine was continued.

Serum sodium rose to 130.0 mmol/L on day 12, 134.0 mmol/L on day 13, and normalized by discharge on day 16 (141.0 mmol/L). In parallel, appetite, spontaneous speech, and psychomotor activity improved within 48–72 h of glucocorticoid initiation, faster than expected from antidepressant efficacy alone. At 4-week outpatient follow-up, serum sodium was 145.0 mmol/L and morning cortisol was 176 nmol/L.

Body weight was recorded at admission (40 kg). Serial inpatient weights were not consistently documented in the chart, but nursing records did not suggest clinically evident fluid overload.

**Table 1:** Key laboratory values and interventions by hospital day

Day	Key events / interventions	Serum sodium (mmol/L)	8 AM cortisol (nmol/L)	ACTH (pg/mL)	CRP (mg/L)
1	Admission; venlafaxine XR 75 mg/day; ChE 4100 U/L (ref 4000–11000)	141.0	112.4	NA	NA
2	Influenza B confirmed	NA	NA	NA	NA
4	Venlafaxine XR increased to 150 mg/day	135.0	NA	NA	55.77
7	Progressive hyponatremia	125.0	NA	NA	10.16
8	Na continued to fall (despite treatment)	120.2	NA	NA	NA
9	Na nadir (despite treatment)	119.4	NA	NA	NA
10	Endocrine reassessment	123.9	122.0	24.4	NA
11	Prednisone acetate 5 mg BID started	NA	NA	NA	NA
12	Clinical improvement	130.0	NA	NA	5.19
16	Discharge	141.0	NA	NA	NA
Follow-up (4 weeks)	4-week follow-up	145.0	176.0 (morning)	NA	NA

Abbreviations: ACTH, adrenocorticotropic hormone; CRP, C-reactive protein; NA, not available; Na, serum sodium

## Discussion and conclusions

This case was revised to avoid single-cause inference and to foreground a pragmatic, multifactorial explanation. The major diagnostic gaps are the lack of urine sodium/osmolality and plasma osmolality, which precludes definitive differentiation among SIADH, hypovolemic hyponatremia, and glucocorticoid-related water-handling impairment. Dynamic confirmation of HPA-axis dysfunction (ACTH stimulation testing) and pituitary MRI were also not performed.

Nevertheless, several observations support relative hypocortisolemia/HPA-axis reserve reduction as a clinically relevant contributor. First, low morning cortisol was present on day 1 before the onset of marked hyponatremia. Second, ACTH was not elevated despite hypocortisolemia and inflammatory stress, consistent with ‘inappropriately normal’ ACTH. Third, the rapid rise in serum sodium and simultaneous improvement in psychomotor activity, speech, and appetite after prednisone initiation, despite continued venlafaxine, provides ex juvantibus support for glucocorticoid-responsive physiology. In acute-care settings where gold-standard tests are unavailable, therapeutic response can strengthen, but not prove, a clinical inference.

SIADH remains a credible alternative or co-mechanism. Influenza B has been associated with SIADH [7], and venlafaxine (an SNRI) can induce or worsen hyponatremia, especially in older women with low body weight [8, 9]. Our patient, a 67-year-old female with BMI 17.8 kg/m^2, fits this high-risk profile. We therefore propose a ‘second-hit’ model: suspected toxic exposure and systemic inflammation may have reduced physiologic reserve, while influenza-related antidiuretic signaling and SNRI up-titration further impaired free-water clearance, producing a refractory phenotype that was unresponsive to routine measures.

From a psychiatric perspective, this case highlights a diagnostic trap. Symptoms of hypocortisolemia (fatigue, anorexia, psychomotor slowing, reduced speech) overlap substantially with depressive deterioration [4–6], potentially prompting escalation of antidepressant doses. When apparent antidepressant non-response co-occurs with hyponatremia, clinicians should broaden the medical differential rather than intensify psychotropics alone. Early electrolyte monitoring and consideration of early-morning cortisol screening may help identify reversible endocrine mimics in medically stressed psychiatric inpatients [10].

Importantly, the patient had a pre-existing depressive disorder and the suicide attempt itself reflects baseline psychiatric vulnerability. We do not argue that suspected OP exposure ‘caused’ depression; rather, hypocortisolemia-related somatic and neuropsychiatric features may have masqueraded as acute depressive worsening during inpatient care, thereby influencing clinical decisions such as SNRI up-titration.

Finally, evidence for OP toxicity was indirect (scene findings, clinical context) and biochemical confirmation was limited by near-normal cholinesterase levels and uncertain dose. Accordingly, our inferences are intentionally cautious and framed as associative and hypothesis-generating.

This case suggests that refractory hyponatremia in psychiatric inpatients can reflect interacting stressors rather than a single etiology. While causality cannot be established, the timeline and glucocorticoid-responsive course support consideration of relative hypocortisolemia/CIRCI within a second-hit model involving infection and SNRI up-titration. Clinicians should consider early-morning cortisol assessment when hyponatremia accompanies sudden deterioration or apparent antidepressant non-response, especially in older, low-BMI patients.

## Data Availability

All data generated or analyzed during this study are included in this published article.

## Abbreviations

ACTH: Adrenocorticotropic hormone
CIRCI: Critical illness-related corticosteroid insufficiency
CRP: C-reactive protein
HPA: hypothalamic-pituitary-adrenal
OP: Organophosphate
SIADH: Syndrome of inappropriate antidiuretic hormone secretion
SNRI: Serotonin-norepinephrine reuptake inhibitor
